# Mitotic centromere-associated kinesin (MCAK): a potential cancer drug target

**DOI:** 10.18632/oncotarget.416

**Published:** 2011-12-31

**Authors:** Mourad Sanhaji, Claire T. Friel, Linda Wordeman, Frank Louwen, Juping Yuan

**Affiliations:** ^1^ Department of Gynecology and Obstetrics, School of Medicine, J. W. Goethe-University, Frankfurt, Germany; ^2^ School of Biomedical Sciences, University of Nottingham, Medical School, Queen's Medical Centre, Nottingham, UK; ^3^ Department of Physiology and Biophysics, University of Washington, Seattle, WA 98195; ^4^ Center for Cell Dynamics, Friday Harbor, Laboratories, Friday Harbor, WA 98250, USA

**Keywords:** MCAK, mitotic kinases, chromosome instability, drug resistance and invasiveness

## Abstract

The inability to faithfully segregate chromosomes in mitosis results in chromosome instability, a hallmark of solid tumors. Disruption of microtubule dynamics contributes highly to mitotic chromosome instability. The kinesin-13 family is critical in the regulation of microtubule dynamics and the best characterized member of the family, the mitotic centromere-associated kinesin (MCAK), has recently been attracting enormous attention. MCAK regulates microtubule dynamics as a potent depolymerizer of microtubules by removing tubulin subunits from the polymer end. This depolymerizing activity plays pivotal roles in spindle formation, in correcting erroneous attachments of microtubule-kinetochore and in chromosome movement. Thus, the accurate regulation of MCAK is important for ensuring the faithful segregation of chromosomes in mitosis and for safeguarding chromosome stability. In this review we summarize recent data concerning the regulation of MCAK by mitotic kinases, Aurora A/B, Polo-like kinase 1 and cyclin-dependent kinase 1. We propose a molecular model of the regulation of MCAK by these mitotic kinases and relevant phosphatases throughout mitosis. An ever-increasing quantity of data indicates that MCAK is aberrantly regulated in cancer cells. This deregulation is linked to increased malignance, invasiveness, metastasis and drug resistance, most probably due to increased chromosomal instability and remodeling of the microtubule cytoskeleton in cancer cells. Most interestingly, recent observations suggest that MCAK could be a novel molecular target for cancer therapy, as a new cancer antigen or as a mitotic regulator. This collection of new data indicates that MCAK could be a new star in the cancer research sky due to its critical roles in the control of genome stability and the cytoskeleton. Further investigations are required to dissect the fine details of the regulation of MCAK throughout mitosis and its involvements in oncogenesis.

## INTRODUCTION: MITOSIS AND CHROMOSOME INSTABILITY

The cell cycle is the series of events that take place in a cell resulting in its DNA replication and division. Numerous mechanisms exist for the control of the cell cycle to ensure smooth and precise progression with high fidelity. Mitosis, the most crucial phase in the cell cycle, has been one of the most active research topics in cell biology since its discovery and accurate description by Walter Flemming [1]. During mitosis replication and division of the nuclear material allows one mother cell to give rise to two daughter cells with exact genetic copies. Spectacular changes occur within the cell during this phase such as chromatin condensation, the nuclear membrane breakdown, mitotic spindle assembly, chromosome congression and chromosome segregation. Correct bipolar spindle formation and attachment of chromosomes to the microtubules (MTs) are prerequisites for the accurate segregation of chromosomes resulting in an errorless chromosome complement in both daughter cells. The inability to faithfully segregate chromosomes in mitosis causes chromosome instability (CIN), a hallmark of solid tumors. It is becoming increasingly clear that CIN is not simply a passenger phenotype but likely plays a causative role in a substantial proportion of malignancies [2]. Moreover, CIN positively correlates with poor patient prognosis, indicating that reduced mitotic fidelity contributes to cancer progression by increasing genetic diversity among tumor cells [3, 4]. Disruption of microtubule dynamics in the mitotic spindle generates mitotic chromosomal instability, commonly caused by the persistent mal-oriented attachment of chromosomes to spindle MTs. Microtubule dynamics, mediated by highly coordinated dynamic growth and shrinkage, governs both chromosome bi-orientation and segregation during cell division [5]. The kinesin-13 family members of MT depolymerizers play essential roles in controlling MT dynamics [6-12].

## MITOTIC CENTROMERE-ASSOCIATED KINESIN (MCAK) AND THE KINESIN-13 FAMILY

Unlike other kinesins, the members of the kinesin-13 family do not use the energy from ATP turnover to move directionally along MTs but, instead, depolymerize them by disassembling tubulin subunits from the polymer end [13, 14]. This family is characterized by the localization of the conserved kinesin motor domain in the middle of the polypeptide [15]. Variable numbers of kinesin-13 family members exist in invertebrate species, such as Klp10A, Klp59C, and Klp59D in *Drosophila melanogaster* [16]. In mammals, three unique genes corresponding to kinesin-13 family members have been identified: Kif2A [17], Kif2B [18] and Kif2C or *m*itotic *c*entromere-*a*ssociated *k*inesin (MCAK) [19]. Kif2A is localized mainly at the centrosome and contributes to bipolar spindle assembly and MT flux, whereas Kif2C/MCAK is found to be localized at the centromere and regulates MT turnover at the kinetochore [6, 11, 16, 20, 21] and also at the plus-ends of interphase and mitotic astral MTs [22-24]. Kif2B has been less well studied. However, it has recently been shown that Kif2B localizes to centrosomes, spindle MTs, kinetochores and the midbody, and is important for spindle assembly, chromosome movement and cytokinesis [8]. Kif24, the fourth member of the kinesin family-13, localizes to centrioles and has recently been implicated in ciliogenesis [25].

MCAK/Kif2C, the founding and best-characterized member of the kinesin-13 family, has an extraordinarily high affinity for MT ends and catalytically destabilizes MTs from either end with a comparable rate [26]. Structurally, MCAK has an N-terminal domain, followed by a positively charged neck, a central catalytic motor domain, and a C-terminal dimerization domain [6]. The catalytic core of MCAK is necessary but not sufficient for depolymerization under physiological conditions and inclusion of the neck domain restores full MT depolymerization activity to the MCAK core motor [27]. Neutralization of the positively charged neck by site-directed mutagenesis markedly reduces enzymatic activity both *in vivo* and *in vitro* [28]. Additionally, the neck is vertically directed toward the surface of the MT between the protofilament groove [14]. More recently, the contribution of the neck domain to MCAK's activity has been precisely studied by applying total internal reflection fluorescence (TIRF) microscopy at the single molecule level: MCAK's positively charged neck enhances its delivery to MT ends by catalyzing the association of MCAK to MTs [29].

Whilst the neck region is important, it is the core motor domain of MCAK that drives MT depolymerization most likely by causing microtubule protofilaments to adopt a depolymerization competent curved conformation. X-ray crystallographic studies show that the MT-binding surface of the core motor adopts a convex form predicted to match the concave shape of a curved MT protofilament [14, 30]. Additionally, MCAK bound with AMPPNP, a non-hydrolysable ATP analogue, stabilizes protofilament curls and rings [31-34]. The currently favored model of how this ability to encourage curving of MT protofilaments translates into depolymerization is that MCAK's ATP turnover cycle, in conjunction with the alteration of this cycle by interaction with the MT, results in MCAK remaining in a weakly bound diffusion competent state whilst on the MT lattice but to switch into a tightly bound depolymerization competent state at or close to the MT end [35]. Here, at the MT end, MCAK can exert its curve inducing effect on the MT protofilaments to best advantage resulting in potent depolymerization activity. In accord with this, an MCAK-decorated bead in the presence of ATP can attach to the MT side, but readily slides along it in either direction under weak external loads. However, the bead is tightly captured by the MT ends and readily causes MT disassembly [36].

While MCAK is found in the cytoplasm throughout the cell cycle, it is highly enriched at centrosomes, centromeres/kinetochores and the spindle midzone during mitosis [19, 37, 38]. In line with this localization, MCAK influences many aspects of mitosis such as spindle assembly, MT dynamics, correct kinetochore-microtubule attachments, and chromosome positioning and segregation [11, 37, 39-41]. Depletion or inhibition of MCAK activity results in improper spindle maintenance and misaligned chromosomes during metaphase in *Xenopus* extract spindles and lagging chromosomes during anaphase [37-39]. Decreased MCAK activity specifically at the centromere leads to poor coordination of chromosome movement, increased kinetochore fiber stability, as measured by acetylated tubulin fluorescence, and increased lagging chromosomes [42]. These effects could be reversed by ectopic anchoring of excess MCAK to centromeres. In addition, analysis of lagging chromatids in mammalian Ptk2 cells depleted of centromeric MCAK shows stretched staining of an autoimmune antibody CREST, a kinetochore marker, indicative of merotelic kinetochore-MT attachment [43]. These erroneous attachments lead to the loss or gain of chromosomes in daughter cells known as “aneuploidy”, which is characteristic of cancer cells [44]. This suggests that MCAK is involved in correcting mal-attachments of kinetochores to MTs prior to anaphase. Thus, precise control of the localization and activity of MCAK is crucial for maintaining genetic integrity during mitosis.

One mechanism by which MCAK is regulated is by association with cofactors such as ICIS (Inner Centromere Kin I Stimulator) [45], hSgo2 [46], EB1 [22, 24, 47] and TIP150 [48]. More interestingly, an increasing body of data is emerging suggesting that the activity and localization of MCAK is regulated via phosphorylation by important mitotic kinases.

## REGULATION OF MCAK BY AURORA B

Aurora B kinase is the catalytic subunit of the chromosome passenger complex (CPC), which contains INCENP, borealin and survivin and is mobile throughout mitosis: the CPC is found at the chromosome arms, the inner centromere and the midzone [49]. The CPC regulates many events in mitosis, including chromosome congression, kinetochore-microtubule attachments, spindle checkpoint control and chromosome segregation, by phosphorylating distinct sets of substrates [50, 51]. The CPC is responsible for recruiting several groups of proteins to the kinetochore/centromere at metaphase: outer kinetochore proteins involved in the spindle assembly checkpoint (SAC) including Mad1, Mad2, Bub1, BubR1, Mps1 and Cenp-E [52-54]; proteins responsible for microtubule-kinetochore interactions, such as Cenp-E, Ndc80, Knl1, Mis12, Zwilch, p150Glued, Dam1 and Plk1 [55-57]; and inner centromeric proteins such as the Shugoshin family proteins Sgo1 and Sgo2 [46, 56, 58]. Thus, the CPC is a critical regulator of centromere/kinetochore functions [59]. Most notably, Aurora B kinase is required for correcting erroneous MT attachments at kinetochores.

In the search for a molecular understanding of the mechanism of action of Aurora B, several groups have focused on the interaction of Aurora B with MCAK. Three high profile studies have shed light on MCAK's regulation by Aurora B [60-62]. Using *in vitro* phosphorylation and mass spectroscopy, several sites in the N-terminus and the neck domain of MCAK have been shown to be phosphorylated by Aurora B [60-62]. Further immunoblotting and immunofluorescence staining confirm T92 in Chinese hamster MCAK [61] and S196 in *Xenopus* MCAK [60] are phosphorylated during mitosis in living cells. Aurora B phosphorylation of MCAK strongly inhibits its ability to destabilize MTs [60-62]. In particular, phosphorylation at S196 appears critical to this activity [60]. Moreover, inhibition of Aurora B activity blocks MCAK accumulation at centromeres [60, 61]. Microinjection of anti-phospho-S196 antibodies causes misalignment of chromosomes at metaphase in *Xenopus* egg extracts and delays chromosome congression to the metaphase plate in cells [61]. Intriguingly, phospho-mimetic MCAK concentrates at the inner centromere, whereas unphosphorylated MCAK prefers a more distal location [61]. These data indicate that Aurora B phosphorylation both positively and negatively regulates MCAK activity by positively influencing the localization of MCAK to the centromere and negatively controlling its MT destabilizing activity. Interfering with this regulation generates defects in spindle structure and chromosome movements in mitosis [60-62]. A fourth important study has further dissected regulation of MCAK by Aurora B, suggesting that Aurora B-dependent chromosome arm and centromere localization is regulated by a distinct two-site phosphorylation mechanisms: T95 phosphorylation facilities MCAK's association with chromosome arms, whereas phosphorylation of S196 causes MCAK to dissociate from chromosome arms and negatively affects its catalytic activity of MCAK [63].

Aurora B is the master regulator of the merotelic resolution pathway that recruits and regulates proteins to correct chromosome-kinetochore mal-attachment [59, 64]. Aurora B is required to release improper MT attachments and MCAK participates in this process [43, 65]. However, how Aurora B-mediated suppression of MCAK activity can contribute to the correction of improper attachments, is counter-intuitive. Several working models have been suggested to explain this issue. It is proposed that the ratio of MCAK/pS196 MCAK is crucial for this function, based on the data that the ratio of MCAK/pS196 MCAK is higher at merotely sites than at properly attached centromeres, implying MCAK is more active at merotely sites [66]. A second interesting idea is that kinetochore-associated MCAK may regulate the attachment status not solely by releasing the attachment, but rather by loosening the MTs ends embedded in the kinetochore to alter MT binding affinity [8, 42]. A third model proposes a so called gradient distribution of Aurora B: when tension across kinetochores is established at metaphase, the extent of the Aurora B activity gradient across sister kinetochore pairs is reduced, inhibition of MCAK by Aurora B is therefore relieved, and active MCAK acts to promote MT dynamics necessary to correct mal-orientated chromosomes [10]. More studies are warranted to precisely delineate how MCAK regulation by Aurora B acts to correct and/or prevent mal-attachments in mitosis.

## REGULATION OF MCAK BY AURORA A

Aurora A, another member of the Aurora kinase family, plays many roles in mitosis mainly related to centrosome functions and spindle assembly. It localizes to centrosomes and spindle poles and drives centrosome maturation, separation and bipolar spindle assembly [67-69]. Aurora A associates with several co-activators including BORA and TPX2 during cell division that dictate its localization, activation and substrate preference [70, 71]. Selective inhibition of Aurora A leads to abnormal mitotic spindles and chromosome segregation defects [72, 73], indicating that Aurora A-associated activity is critical for spindle formation and spindle dynamics during mitosis.

Interestingly, several reports associate Aurora A with MCAK function and localization. In mitotic U2OS cells in the absence of Aurora A, MCAK is decreased at spindle poles, whereas ch-TOG (colonic hepatic tumor-overexpressed gene), a functional antagonist of MCAK, is increased in mitotic U2OS cells, leading to extra-poles formation [74]. It has also been shown, using *Xenopus* egg extracts to form spindles in the absence of chromatin and centrosomes, that Aurora A controls MCAK's localization and activity [75]. This regulation is important to focus MTs at aster centers and to facilitate the transition from asters to bipolar spindles. Additionally, MCAK co-localizes with NuMA and XMAP215 at the center of Ran asters, where its activity is regulated by Aurora A-dependent phosphorylation of S196, which contributes to proper pole focusing. MCAK localization at spindle poles is also controlled via S719 phosphorylation by Aurora A, which positively enhances bipolar spindle formation. This study suggests that Aurora A targets MCAK to spindle poles via phosphorylation on S719, and regulates its activity by phosphorylation at S196. It is however unclear how this phosphorylation of MCAK by Aurora A directs its localization. These results indicate that Aurora A regulates the localization of MCAK at spindle poles and that MCAK is involved in spindle pole integrity. However, the molecular mechanism by which Aurora A impacts MCAK's localization has not been described. Furthermore, whether this regulation of MCAK by Aurora A also affects its catalytic activity remains undefined. Nevertheless, as described above, the localization and activity of MCAK at the centromere/kinetochore are controlled by Aurora B kinase, whereas the localization and activity at spindle poles appear to be regulated by Aurora A.

## REGULATION OF MCAK BY CYCLIN-DEPENDENT KINASE 1 (CDK1)

Cdk1/cyclin B1 is a kinase essential for the initiation of mitosis. We have found that the phenotype of depleting cyclin B1, the regulatory subunit of Cdk1, is reminiscent of that of the inhibition of MCAK. Moreover, MCAK and Cdk1 co-localize at centrosomes and they are associated with each other in mitosis. Further work shows that Cdk1/cyclin B1 regulates the function and localization of MCAK by phosphorylating T537 in the core domain [76]. This phosphorylation of MCAK by Cdk1/cyclin B1 attenuates its MT-destabilizing activity *in vitro* and *in vivo*. Phosphorylation of MCAK by Cdk1 promotes the release of MCAK from centrosomes and is required for proper spindle formation. Furthermore, interfering with the regulation of MCAK by Cdk1 causes dramatic defects in spindle formation and in chromosome positioning. Unlike Aurora B, Cdk1 phosphorylates only one residue T537 in the core domain of MCAK. T537 is located in the L12 loop, which is immediately C-terminal to the α4 helix of the core domain in MCAK. It has been suggested that the α4 helix is directly involved in binding to a curved conformation of tubulin at the ends of MT protofilaments and thereby facilitates depolymerization [14]. It is therefore possible that the introduction of a negative charge adjacent to the α4 helix via phosphorylation of T537, could disrupt the interaction of MCAK with the MT end, thus causing attenuation of MCAK's MT-destabilizing activity. Further investigations are required to define precisely how MCAK is coordinated and controlled by Cdk1/cyclin B1 at centrosomes/spindle poles.

## REGULATION OF MCAK BY POLO-LIKE KINASE 1(PLK1)

Five mammalian Plk family members have been identified to date, Plk1-5 [77]. Plk1, the best studied member of the family [78], is a key regulator of cell division in eukaryotic cells. Plk1 controls multiple events in mitosis such as centrosome maturation, bipolar spindle formation, stable microtubule-kinetochore attachment, cohesion dissociation, chromosome alignment and segregation, and cytokinesis [79, 80]. In accord with its diverse functions, the localization of Plk1 during mitosis is dynamic. Plk1 first associates with centrosomes in prophase before it localizes to spindle poles and kinetochores in prometaphase and metaphase. In anaphase, Plk1 is recruited to the central spindle and finally accumulates at the midbody in telophase. Proteomic studies using oriented peptide libraries have shown that the polo-box binding domain (PBD) at the C-terminus of Plk1, dictates the localization of this kinase to cellular structures [81, 82]. This domain binds to specific phosphorylated sequence motifs that are created by other priming kinases or are self-primed by Plk1 itself, thus providing an efficient mechanism to regulate localization and substrate selectivity in time and space [83, 84]. Thus, the PBD provides a much more compelling site to specifically inhibit Plk1 [85, 86]. Plk1 is a proliferation marker and highly expressed in a broad spectrum of human tumors, which is associated with prognosis of tumor patients and suggestive of its involvement in oncogenesis [77, 87, 88]. Interestingly, Plk1 expression is affected by several drugs, like metformin [89]. Despite intensive investigations, the role of the multifaceted Plk1 in oncogenesis remains incompletely understood at the molecular level.

It has been revealed that Plx1, the analogue of Plk1 in *Xenopus*, phosphorylates the N-terminal region (aa 2-116) of MCAK in kinase assay *in vitro* [90]. Moreover, priming MCAK by Plx1 produced robust phosphorylation on T95 site but little on S196 in MCAK by Aurora B, suggesting that this is the key event to allow Aurora B to phosphorylate the residue T95 [90]. Since T95 phosphorylation is critical for MCAK's localization on chromosome arms in prophase and prometaphase [63], the data suggest that Plx1 could be the priming kinase for Aurora B to promote the localization of MCAK to the chromosome arms. This is an interesting finding indicative of a collaborative action of Plk 1 and Aurora B in the regulation of MCAK. It remains to be investigated which residue in the N-terminus of MCAK is phosphorylated by Plk1 and how this modification facilitates phosphorylation of T95 by Aurora B. It is also necessary to define if the same takes place *in vivo* and whether this collaboration model could also be valid for mammalian cells.

A second study dealing with MCAK's regulation by Plk1 has recently been reported [91]. Based on the data from *in vitro* phosphorylation assay and mass spectrometry, six serines (S592, S595, S621, S632, S633 and S715) at the C-terminus of MCAK have been identified as substrates of Plk1. In contrast to regulation by Aurora A/B and Cdk1, phosphorylation of MCAK by Plk1 stimulates the MT depolymerization activity of MCAK in cells. Overexpression of a Plk1 phosphomimetic MCAK mutant causes a dramatic increase in misaligned chromosomes and in multipolar spindles in mitotic cells, while overexpression of a non-phosphorylatable MCAK mutant results in defects in anaphase with sister chromatid bridges. These data imply that the enhanced enzymatic activity of MCAK by Plk1 is required for correction of mal-attachment of MTs. On the other hand, a temporal dephosphorylation of MCAK is of importance for proper chromosome alignment and bipolar spindle formation. This study also suggests that phosphorylation of MCAK by Plk1 may alter its molecular conformation. Further investigations are warranted to elucidate the structural basis of this potential Plk1-induced MCAK conformational change. Moreover, it will be interesting to identify the major phosphorylation site in MCAK's C-terminus by Plk1 and to dissect the function of each phosphorylation site. It may be that Plk1 acts in a similar way as Aurora B phosphorylates different residues in MCAK controlled both temporally and spatially to coordinate MCAK's function at various stages of mitosis. Furthermore, since both the N-terminus in *Xenopus* and the C-terminus in mammalian cells are phosphorylated by Plx1 and Plk1, respectively, it remains possible that both regions of MCAK are regulated by Plk1 at different stages *in vivo*. It is tempting to speculate that the regulation of MCAK by Plk1 will exhibit a more complex picture, even than Aurora B, with various functions depending on different subcellular locations and on different time points in mitosis. Moreover, Santamaria and colleagues have investigated the Plk1-dependent phosphoproteome of the human mitotic spindle using an elegant method of isotope labeling of amino acids in cell culture [92]. One of the most interesting findings is that MCAK's spindle association is highly dependent on Plk1 activity [92]. It remains to be explored if Plk1 regulates this association directly or indirectly.

## ORCHESTRATED REGULATION OF MCAK BY MITOTIC KINASES/PHOSPHATASES

**Figure 1:**
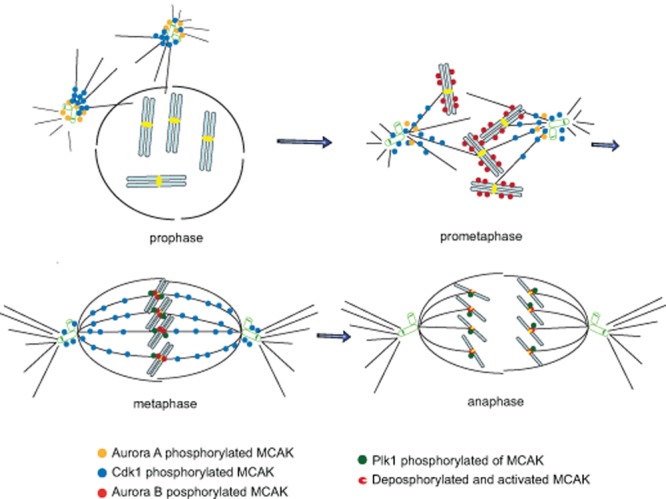
Scheme of MCAK regulation by mitotic kinases and phosphatases Current data suggest that MCAK undergoes complex spatiotemporal regulation by mitotic kinases Aurora A/B, Plk1 and Cdk1/cyclin B1. In early mitosis, S196 phosphorylation of MCAK by Aurora A reduces its activity and facilitates the transition from asters to bipolar spindles, whilst MCAK localization at spindle poles is regulated through another Aurora A phosphorylation site S719 and positively enhances bipolar spindle formation. Cdk1 phosphorylates T537 in the core domain of MCAK, attenuates its activity, and drives MCAK from spindle poles to other locations and promotes proper spindle formation. Thus, the localization and activity of MCAK at centrosomes and spindle poles appear to be mainly controlled by coordinated regulation of Aurora A and Cdk1. In prometaphase, the localization of MCAK to the chromosome arms is controlled by Aurora B, possibly supported by Plk1. In metaphase, the localization and activity of MCAK at the centromeres and kinetochores are finely regulated via phosphorylation by Aurora B. By contrast, Plk1 promotes the activity of MCAK at kinetochores. Co-ordinated regulation of MCAK by Aurora B and Plk1 might fine-tune its activity for correction of mal-attachments. Finally, in anaphase, the activity of MCAK is further coordinated and controlled by Aurora B and Plk1, possibly balanced by phosphatases.

During the cell division cycle, mitotic entry, centrosome separation, spindle assembly, chromosome congression/segregation, and cytokinesis must all be tightly coordinated to ensure that the two daughter cells inherit the same genetic material. Central to this coordination are several protein kinases including Cdk1, Plk1, Aurora A and Aurora B, which regulate the functions of many molecules in a precisely coordinated and finely tuned manner. Current data suggest that MCAK undergoes complex spatiotemporal regulation by these critical mitotic kinases throughout mitosis (Fig. 1). In early mitosis, the localization and activity of MCAK at centrosomes and spindle poles appear to be mainly controlled by the coordinated regulation of Aurora A and Cdk1. S196 phosphorylation of MCAK by Aurora A reduces its activity and facilitates the transition from asters to bipolar spindles, whilst MCAK localization at spindle poles is regulated through another Aurora A phosphorylation site S719 and positively enhances bipolar spindle formation. Cdk1 phosphorylates T537 in the core domain of MCAK, reducing its activity, and driving MCAK from the spindle poles to other locations, thus promoting proper spindle formation. In prometaphase, the localization of MCAK to the chromosome arms seems to be controlled by Aurora B, possibly aided by Plk1. In metaphase, the localization of MCAK at the centromeres and kinetochores is finely regulated via phosphorylation by Aurora B. In this process, the activity of MCAK at the centromeres/kinetochores is potentially positively promoted by Plk1-mediated activity to fine-tune the regulation by Aurora B. This coordinated regulation may allow efficient correction of mal-attached microtubule-kinetochore. Finally, in anaphase, the activity of MCAK is controlled by Aurora B and Plk1, balanced possibly by phosphatases. The picture of MCAK regulation by mitotic kinases (Fig. 1) is still immature and more studies are needed to complete the picture. The final picture displaying the temporal and spatial regulation of MCAK in mitosis may be more complex than we had previously imagined.

Specific phospho-antibodies targeting each phosphorylation site by each mitotic kinase will be of great use in deciphering when, where and by which kinase MCAK is phosphorylated throughout various mitotic stages. The specific small molecule compounds targeting Cdk1, Aurora A, Aurora B or Plk1 will also be useful to uncover the timing and location of each phosphorylation, and to study the exact impact of each kinase on MCAK in mitosis. Moreover, current data imply that timely dephosphorylation is also necessary for the proper mitotic function of MCAK, as phospho-mimetic forms of MCAK induce many defects in mitosis [76, 91]. Recent work has exposed the conserved serine-threonine phosphatases PP1 and PP2A as key regulators of various mitotic processes. PP1 is known to both localize to kinetochores and to reverse phosphorylation generated by Aurora B [55], indicating that PP1 is a prime candidate for opposing Aurora B-dependent maintenance of kinetochore integrity. Indeed, kinetochore disassembly following Aurora B inhibition is prevented by inhibiting PP1 [55]. Thus, the proper regulation of MCAK at the centromeres/kinetochores likely depends on a biased turnover between kinases and their counteracting phosphatases. Intensive investigations are required to understand this network of regulation. It is also of importance to determine if interaction partners of MCAK are regulated by the same mitotic kinases, which will further dissect the molecular network of MCAK regulation in mitosis. Finally, there remains much work still to do to define the functional relationship among the three members of the Kinesin-13 family found in mammalian cells and also with members of other kinesin families, such as the family-14 and family-8 [93, 94].

## MCAK IN ONCOGENESIS: ASSOCIATION OF MCAK WITH CANCER DEVELOPMENT

MCAK is important for proper spindle formation, correction of aberrant attachments of microtubule-kinetochore and for chromosome segregation. To accomplish this, the activity and localization of MCAK must be closely regulated by various kinases and phosphatases in a finely orchestrated manner. Unfortunately, mitotic kinases and phosphatases can become unregulated resulting in abnormal mitosis, chromosome instability and ultimately transformation. By the same logic deregulation of MCAK may play a role in cancer development.

Indeed, it has been reported that MCAK is one of dozens of trans-activated genes in a genome-wide expression analysis of 81 breast cancer tissues by means of a combination of cDNA microarray and laser microbeam microdissection [95]. Further analysis demonstrated that MCAK is overexpressed in primary breast cancer tissues as well as in cell lines [96, 97]. In addition, MCAK expression is significantly suppressed by ectopic introduction of p53 [97], suggesting that highly expressed MCAK might be involved in breast cancer development. This elevated expression of MCAK is observed not only in breast cancer but also in gastric cancer [98], and enhanced MCAK expression is significantly linked to lymphatic invasion, lymph node metastasis and poor prognosis in gastric cancer patients [98]. Furthermore, this observation is underscored by a study based on 120 colorectal cancer samples: MCAK expression is significantly higher at both the mRNA and protein levels, compared to paired corresponding normal tissues, and this elevated expression level is markedly correlated with lymph node metastasis, venous invasion, peritoneal dissemination, Dukes' classification and poor survival rate [99]. Recently, this observation has been further strengthened by a study containing 176 samples derived from colorectal, pancreatic, gastric, breast and head and neck cancer tissues [100]. Comparing expression levels among cancer types, it is noted that MCAK is most strongly overexpressed in gastric, breast and colorectal cancer and less pronounced in pancreatic and head and neck cancer. More recently, MCAK gene has been found to be highly expressed in glioma samples, which is associated with histopathological grades [101]. Taken together, the data highlight that MCAK is aberrantly regulated in cancer cells and enhanced MCAK levels are associated with cancer progression, invasiveness, metastasis and poor prognosis, particularly in breast, gastric and colorectal cancer.

Deregulated MCAK results in defects in spindle formation and chromosome segregation, which lead to further chromosomal instability. Being capable of escaping apoptosis and surviving such defects, cancer cells proliferate regardless of chromosomal instability, promoting progression of cancer cells. As described above, it seems p53 is involved in regulating the expression of MCAK [97]. Further studies are required to corroborate the relationship between p53 status and the MCAK level in primary cancer tissues. More questions have to be addressed: Does p53 directly affect the transcriptional activation of the MCAK promoter, or indirectly via other transcriptional factors? As microRNAs (miRNAs) are becoming more and more important in regulating mRNA expression of key molecules, is miRNA involved in the regulation of MCAK mRNA in cancer cells? Or are elevated levels of MCAK more ascribed to deregulated protein turnover? Intriguingly, the data suggest that enhanced MCAK is correlated not only with progression but also with invasiveness/metastasis in cancer cells, suggesting MCAK is possibly also involved in the alteration of cell motility in cancer cells. Cell motility is a complex process requiring coordinated organization of actin and MT cytoskeletons in physiological and pathological conditions such as cancer cell metastasis. It will be interesting to understand how deregulated MCAK in cancer cells promotes migration/invasiveness/metastasis: Is elevated MCAK able to reorganize MT cytoskeleton and to alter the motility of cancer cells, in particular, in non-proliferating cancer cells? How do changes in MCAK levels influence tubulin expression and auto-regulation? Is deregulated MCAK associated with remodeling the environments of cancer cell including cell-cell and cell-extracellular matrix adhesion? In addition, early steps in metastasis are often linked with epithelial-mesenchymal transition (EMT), a process that allows polarized epithelial cells into isolated, migratory cells with mesenchymal morphology and characteristics [102]. Does deregulated MCAK facilitate EMT by reorganizing cell cytoskeleton, possibly coordinated with other molecules/signal pathways? Does the front line/part of cancer tissues express more MCAK? Numerous questions await answers.

It is intriguing to note that highly expressed MCAK is linked with invasiveness and metastasis in colorectal cancer [99]. It is known that more than 80% of colorectal cancers have inactivating mutations in the adenomatous polyposis coli (APC), a tumor suppressor linked to the initiation and progression of colon cancer [103, 104]. APC participates to the Wnt signaling pathway by downregulating β-catenin and controlling gene transcription and cell proliferation. Moreover, APC plays a key role in directed cell migration by showing its regulated localization during cell migration and the ability to bind multiple polarity proteins and MT-associated molecules [105]. Interestingly, the APC protein mediates direct interactions with MTs and the MT plus-end tracking protein EB1 (end-binding protein 1), which promotes MT growth through increased rescue frequency and decreased catastrophe of plus-ends [106]. Interestingly, MCAK has also been reported to track MT plus-ends [22] and to co-localize with EB1 at growing MT ends [23]. It will be interesting to delineate how the plus-end tracking proteins function in colon cancer cells, with inactive APC and elevated MCAK, in context of the dynamics of MT cytoskeleton and cell motility.

## INVOLVEMENT OF MCAK IN DRUG RESISTANCE

Overexpression of MCAK is not only associated with malignance progression, but also with drug resistance. Taxanes, used either as single agents or in combination with multiple other anticancer agents, are routinely used for a wide range of solid tumors [107]. Despite their widespread use, the clinical effectiveness of taxanes is hampered by its severe side-effects and its resistance, which ultimately leads to relapse and poor prognosis. Various mechanisms have been implicated in acquired or secondary taxane resistance [108]. It is reported that overexpression of MCAK confers resistance to paclitaxel and epothilone A [109]. It is further demonstrated that paclitaxel resistant cells resulting from MCAK overexpression displays a decrease in MT polymer and an increase in the frequency of MT detachment from centrosomes [109]. Moreover, loss of MCAK reverses this aberrantly high frequency of MT detachment and increases their sensitivity to paclitaxel [110]. The results indicate that MCAK affects cell sensitivity to paclitaxel by modulating MT morphology and dynamics.

## MCAK AS A POTENTIAL MOLECULE TARGET FOR CANCER THERAPY

MCAK is also identified as a novel cancer antigen, suggesting the possibility of cancer specific immunotherapy [98]. This notion is underscored by a recent observation that MCAK is capable of inducing spontaneous T cell responses *in vivo* resulting in highly functional MCAK-specific T cells in both patients with colorectal cancer and healthy donors [100]. MCAK serves as an antigen is further supported by another study showing MCAK peptides are able to induce cytotoxic T lymphocytes to lyse cancer cells in an HLA-A2- or HLA-A24-restricted manner [111]. Together, the data imply that MCAK is possibly a promising target for cancer immunotherapy for colorectal and gastric cancers.

**Figure 2:**
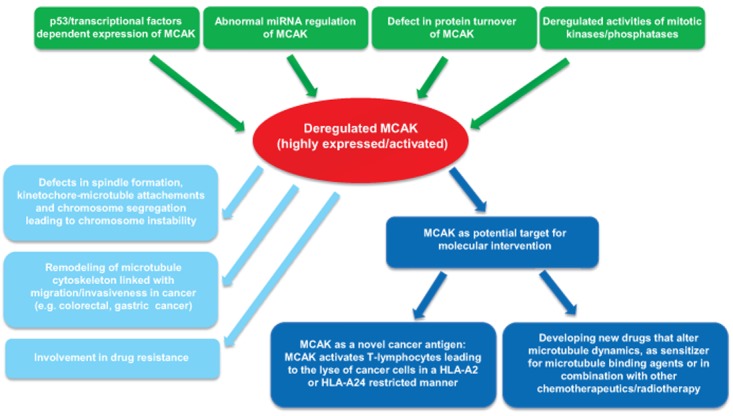
Summary of MCAK's involvements in oncogenesis In gastric, colorectal and breast cancer, MCAK is overexpressed, which could be contributed by defects in promoter control, mRNA stability and protein turnover, possibly associated with deregulated kinases/phosphatases in cancer cells. The highly expressed MCAK could result in abnormal spindle formation, erroneous attachment and failure in chromosome segregation, leading to chromosomal instability and promoting cancer progression. Enhanced MCAK is linked to invasiveness/metastasis of cancer patients, which could be caused by remodeling MT cytoskeleton and altering cell shape and migration. Elevated MCAK could reorganize MT morphology/dynamics and contribute to resistance of the MT binding agents. In addition, MCAK could be considered as a potential target for molecular intervention: either as a novel antigen, provoking immunoreaction of cancer patients, or as a MT regulator/modulator, in combination with other chemotherapeutic drugs.

Targeting mitotic kinesins, such as Eg5, has been regarded as a promising strategy for cancer therapy [112]. The unique ability of MCAK to regulate MT dynamics makes it a potential target for development of new drugs that alter spindle function [113]. It has been shown that malignant cell lines are more sensitive to depletion of MCAK, in comparison with normal cells. In addition, MT interfering drug paclitaxel or vinblastine induces more cytoskeleton defects in HeLa cells depleted of MCAK [113]. Moreover, using quantitative immunofluorescence and fluorescence recovery after photobleaching, the differences in spindle organization is analyzed in cells treated with low levels of paclitaxel, or with MCAK inhibition [114]. Interestingly, paclitaxel treatment causes a disruption in spindle MT organization marked by a significant increase in MTs near the poles and a reduction in K-fiber fluorescence intensity, whereas MCAK inhibition triggers a dramatic reorganization of spindle MTs with a significant increase in astral MTs and reduction in K-fiber fluorescence intensity [114]. Moreover, MCAK depletion promotes dramatic spindle rocking in early anaphase, and this effect is also observed with taxol treatment [115], indicative of defects in cytokinesis. These data support the idea that combination of MCAK suppression with paclitaxel perturbs synergistically spindle organization, which could induce severe irreversible mitotic defects, extending mitotic timing and leading further to mitotic catastrophe and apoptosis in cancer cells. These studies suggest that MCAK might be a good target for new drug development, which could be particularly useful in combination with currently available anti-microtubule agents. In fact, it is reported that one form of sulfoquinovosylacylglycerols (SQAGs) targets the activity of MCAK in cells [116]. It will be interesting to examine if the p53 status and genome instability influence its effectiveness [117, 118]. Figure 2 illustrates the involvement of deregulated MCAK in tumor development, invasiveness/metastasis, drug resistance, and the potential for MCAK as a novel target.

## CONCLUSIONS AND OUTLOOK

The finely tuned regulation of MCAK by various mitotic kinases and phosphatases is essential for the faithful segregation of chromosomes in mitosis and for safeguarding genome stability. Current data suggest MCAK undergoes complex spatiotemporal regulation during mitosis mainly by Aurora B, coordinated by other critical mitotic kinases Aurora A, Plk1 and Cdk1/cyclin B1. Further investigations are required to define the precise cross-talk networks among these kinases throughout mitosis and their balance by phosphatases. MCAK expression is deregulated in breast, gastric and colon cancer, which is highly correlated with cancer progression, invasiveness and metastasis. However, the molecular mechanisms, which drive high expression of MCAK in those cancers, are not clear. It will be interestingly to explore the signal pathways, by which suppression of MCAK renders resistant cancer cells re-sensible to taxanes. It is also important to investigate how overexpression of MCAK increases mobility in cancer cells and promotes invasiveness and metastasis. In addition, it will be of interest to examine whether MCAK could indeed serve as a new target for molecular intervention, as an antigen for immunotherapy, or as a mitotic regulator in combination with other agents interfering with mitosis. It will be also of clinical importance to study the correlation between abnormal activities of mitotic kinases and deregulated MCAK activity in primary cancers.
